# Functional Characterization of the Poplar R2R3-MYB Transcription Factor PtoMYB216 Involved in the Regulation of Lignin Biosynthesis during Wood Formation

**DOI:** 10.1371/journal.pone.0076369

**Published:** 2013-10-28

**Authors:** Qiaoyan Tian, Xianqiang Wang, Chaofeng Li, Wanxiang Lu, Li Yang, Yuanzhong Jiang, Keming Luo

**Affiliations:** 1 Key Laboratory of Eco-environments of Three Gorges Reservoir Region, Ministry of Education, Chongqing Key Laboratory of Transgenic Plant and Safety Control, Institute of Resources Botany, School of Life Sciences, Southwest University, Chongqing, China; 2 College of Horticulture and Landscape Architecture, Southwest University, Chongqing, China; 3 Key Laboratory of Adaptation and Evolution of Plateau Biota, Northwest Institute of Plateau Biology, Chinese Academy of Sciences, Xining, China; National Taiwan University, Taiwan

## Abstract

Because of the importance of wood in many industrial applications, tremendous studies have been performed on wood formation, especially in lignin biosynthesis. MYB transcription factors (TFs), which consist of a large family of plant TFs, have been reported to directly regulate lignin biosynthetic genes in a number of plants. In this study, we describe the cloning and functional characterization of *PtoMYB216*, a cDNA isolated from Chinese white poplar (*Populus tomentosa* Carr.). *PtoMYB216* encodes a protein belonging to the R2R3-MYB family and displays significant similarity with other MYB factors shown to regulate lignin synthesis in *Arabidopsis*. Gene expression profiling studies showed that *PtoMYB216* mRNA is specifically expressed during secondary wall formation in wood. The 1.8-kb promoter sequence of *PtoMYB216* was fused to the GUS coding sequence and introduced into wild-type *A. thaliana*. GUS expression was shown to be restricted to tissues undergoing secondary cell wall formation. Overexpression of *PtoMYB216* specifically activated the expression of the upstream genes in the lignin biosynthetic pathway and resulted in ectopic deposition of lignin in cells that are normally unligninified. These results suggest that *PtoMYB216* is specific transcriptional activators of lignin biosynthesis and involved in the regulation of wood formation in poplar.

## Introduction

The secondary cell wall in higher plants consists mainly of cellulose, lignin and xylan. Lignin is the second most abundant plant biopolymer mainly present in the secondary walls in wood, which allowing mechanical support and efficient conduction of water and solutes over long distances within the vascular system. Lignin is a polymer of complex phenylpropanoid compounds formed by three monolignols, including ρ-coumaryl alcohol, coniferyl alcohol and sinapyl alcohol, which give rise to p-hydroxyphenyl (H), guaiacyl (G), and syringyl (S) [1]. The biosynthetic pathway of monolignols is involved in the general phenylpropanoid pathway leading to the production of hydroxycinnamoyl CoA esters, which are the common precursors of diverse groups of chemical compounds, such as flavonoids, suberin, coumarins, quinones and lignin. The lignin polymer is mainly deposited in the tracheary elements and fibers, and also found in other cell types or tissues, such as the endodermis, periderm and epidermis of some plant species, which confers stable and protective coatings to protect the secondary walls from physical and biological attacks and provide rigidity and impermeability [2]. The lignin polymer constitutes the first line of defense against biotic and abiotic stresses, which resistance to wounding, ultraviolet light irradiation and pathogen attack [3], [4].

To date, lignin biosynthetic pathway has been well determined and many proteins catalyzing deposition of lignin and polysaccharides during secondary cell wall formation have been characterized [5]. The first key enzyme in lignin biosynthetic process is the L-phenylalanine ammonia-lyase (PAL) which catalyzes a deamination of phyenylalanine to produce cinnamic acid [6]. Cinnamic acid is hydroxylated by cinnamate 4-hydroxylase (C4H) to generate ρ-coumaric acid [7], which is converted to ρ-coumaroyl-CoA by 4-coumarate: CoA ligase (4CL) [8]. This product proceeds through a series of transformation into monolignol by the action of caffeoyl-CoA O-methyltransferase (CCoAOMT) [9], ferulate 5-hydroxylase (F5H) [10] cinnamoyl-CoA reductase (CCR) [11], and cinnamoyl alcohol dehydrogenase (CAD) [12], respectively.

Recent studies have demonstrated that formation of secondary wall requires a coordinated transcriptional activation of the genes involved in the lignin biosynthesis [1], [13]. Many transcription factors, belonging to NAC, MYB, and WRKY gene families, have been shown to regulate lignin biosynthetic pathway in various plant species [14], [15]. Due to the difficulty of genetic studies of gene functions in tree species, most of these wood-associated transcription factors have not yet been subjected to functional characterization. To date, most lignin activators reported are from the MYB family, particularly the large family of R2R3-MYB [16]. Indeed, a number of R2R3 MYB proteins have been confirmed in the regulation of phenylpropanoids biosynthesis, such as flavonoids [17], [18], anthocyanin [19], [20], and lignins [21], [22]. Some of these MYB transcription factors have been shown to regulate the entire phenylpropanoid metabolism, and the others were proposed to specifically regulate the lignin biosynthesis. The first identified lignin-specific transcription factors were AtMYB46, AtMYB83, AtMYB58 and AtMYB63 from *Arabidopsis thaliana* [23], [24], [25], which activated the entire secondary wall biosynthesis. AtMYB83, a homology of AtMYB46, is not only lignin pathway regulators, but also redundantly activate the entire process of secondary cell wall formation [5], [26]. Some of R2R3 MYBs isolated from other species were also demonstrated to mediate the regulation of the monolignol pathway. Among these transcription factors are PtMYB1, PtMYB4 and PtMYB8 from *Pinus taeda* [21], [27], EgMYB2 from *Eucalyptus gunnii* [22], [28] and PtrMYB3 and PtrMYB20 from *Populus trichocarpa* [29]. These pine and eucalyptus MYB genes, which are homologs of AtMYB46 and AtMYB83, have been shown to cause ectopic deposition of lignin or altered phenylpropanoid metabolism when overexpressed in tobacco or spruce [21], [22], [27]. In *Arabidopsis*, most of monolignol genes are directly regulated by AtMYB58 and AtMYB83 through AC elements that are commonly present in the promoters of lignin biosynthetic genes, except for F5H [25]. Therefore, regulation by lignin activators is global rather than specific for certain pathway genes. However, it is not clear whether these wood-associated transcription factors also regulate the biosynthesis of other secondary wall components during wood formation.

Worldwide, poplar is an economically important woody biomass because of its high growth capacity, adaptability to varied environments and the various uses in industry, such as pulping and paper-making. Lignin is the second most abundant plant biopolymer in vascular plants, and accounts for 30% of the dry weight in poplar. Although the lignin-related MYB transcription factors in *Arabidopsis* have been studied well, most of the wood-associated transcription factors have not yet been subjected to functional characterization due to the difficulty of genetic studies of gene functions in tree species. In a previous study, detailed annotation and phylogenetic analysis of the entire R2R3-MYB family encoded in the *Populus* genome have been performed [30]. Many of the *Populus* R2R3-MYB proteins implicated in the regulation of genes encoding lignin biosynthetic enzymes are divided into a specific clade. This clade also includes AtMYB46 [5], *PtMYB4* [21], EgMYB2 [22] and PgMYB4 [31], which alter the accumulation of transcripts corresponding to genes encoding lignin biosynthetic enzymes. The transcript abundance profile also suggests that, like their counterparts in other plant species, these *Populus* transcription factors function in xylem-based processes, perhaps regulating genes encoding enzymes of the lignin biosynthetic pathway [30]. In this study, we isolated a wood-associated MYB transcription factor, PtoMYB216, from Chinese white poplar (*P. tomentosa* Carr.). Phylogenetic analysis showed that PtoMYB216 has a closely relationship with AtMYB61, AtMYB83, AtMYB46 and EgMYB2. PtoMYB216 was able to activate the biosynthetic pathways of lignin, suggesting that PtoMYB216 is involved in the regulation of the lignin biosynthetic pathway in poplar.

## Materials and Methods

### Plant Materials

*Populus tomentosa* Carr. (clone 73) is grown in the greenhouse at 25°C under a 14-/10-h light/dark cycle with supplemental light (4500 lux). Wild-type *Arabidopsis thaliana* (ecotype Columbia) were grown in a greenhouse. The temperature was maintained at 22–23°C and the photoperiod was 16 h.

### Cloning of *PtoMYB216*

The cDNA fragment encoding *PtoMYB216* was amplified with gene-specific primers (PtoMYB216-F: 5′-GTTGCCAGCACTGCATTGCAG-3′; PtoMYB216-R: 5′-GTGAACCTATATGGATTGAC-3′) based on *PtrMYB216* (JQ801749) from *P. trichocarpa* by polymerase chain reaction (PCR). The PCR reaction was carried out with pfu DNA polymerase (Takara, Dalian, China) in a total volume of 50 µl at 94°C for 3 min; 32 cycles of 94°C for 45 s, 54°C for 45 s and 72°C for 90 s, followed by a final extension of 72°C for 10 min. The sequence of *PtoMYB216* was deposited in GenBank under accession number JQ801749. The PCR product was cloned into the plant binary vector pCXSN [32]. The resulting vector p*35S:PtoMYB216*, with the *PtoMYB216* open reading frame driven by the cauliflower mosaic virus (CaMV) 35S promoter and the hygromycin phosphotransferase (HPT) gene, which confer resistance to hygromycin, were transferred into *Agrobacterium tumefaciens* strains EHA105 by the freeze-thaw method.

### Sequence Comparisons and Phylogenetic Analysis

The deduced amino acid sequences were analyzed using the program DNAMAN and the software MEGE version 4.1 (Lynnon Biosoft, Quebec, Canada). Amino acid sequence alignments were performed with DNAMAN software. The phylogenetic tree of lignin-associated MYBs was constructed by the neighbor-joining method for *P. trichocarpa* and other plants using MEGA 4.1 software.

### Transformation of *P. tomentosa* Carr. Plants

*A. tumefaciens* strains EHA105 containing p*35:PtoMYB216* were incubated in liquid yeast extract peptone medium supplemented 100 µmol/L acetone-syringone at 18°C with constant shaking (200 rpm) until the culture reached an optical density of 0.8 at OD 600 nm. The *A. tumefaciens* culture was then diluted with one volume of liquid woody plant medium (WPM) [33].

Poplar transformation methods were described previously by Jia et al [34]. Leaves of Chinese white poplar (*P. tomentosa* Carr.) were excised from in vitro plantlets, cut into disks and dipped in the diluted *Agrobacterium* culture for 8–10 min. After excess liquid on the surfaces was absorbed by sterilized paper, the leaf disks were transferred to WPM medium (2.0 mg/L zeatin, 1.0 mg/L 1-naphthalene acetic acid [NAA]). The infected disks were co-cultivated in the dark for 2 days and then transferred to callus-inducing medium containing 2.0 mg/L zeatin, 1.0 mg/L NAA,400 mg/L cefotaxime, 9 mg/L hygromycin and 0.8% (w/v) agar. After 2–3 weeks of culture in the dark, these leaf disks with induced calli were subcultured on screening medium (2.0 mg/L zeatin, 0.1 mg/L NAA, 400 mg/L cefotaxime, 9 mg/L hygromycin and 0.8% (w/v) agar) to induce adventitious buds. Regenerated shoots were transferred to rooting medium, containing 0.1 mg/L NAA, 400 mg/L cefotaxime and 9 mg/L hygromycin. Transgenic plants were selected with 9 mg/L hygromycin. Rooted plantlets were acclimatized in pots placed inside a humid chamber (16 h photoperiod, 25°C, 70% relative humidity) for 2 weeks and finally transferred to the greenhouse.

### Molecular Analysis of Transgenic Plants

Genomic DNA was extracted from poplar leaf material (300 mg) of untransformed control and transgenic plants using a CTAB method [34]. Each PCR-mixture (10 µl) contained 5.5 µl GoTaq^R^ Green Master Mix (Promega, Beijing, China), 0.25 µl each primer, 0.5 µl cDNA, 3.5 µl Nuclease-Free water. Polymerase chain reaction analysis was carried out employing gene-specific primers: HPT (F: 5′-ATCGGACGATTGCGTCGCATC-3′; R: 5′-GTGTCACGTTGCAAGACCTG-3′). Polymerase chain reaction conditions were 94°C for 3 min; 32 cycles of 94°C for 30 s, 56°C for 30 s, 72°C for 1 min, followed by a final extension of 72°C for 10 min. Polymerase chain reaction products were resolved on a 1% (w/v) agarose gel and visualized after ethidium bromide staining.

### Transformation of *A. thaliana*

The GUS reporter gene was employed to study the developmental expression pattern of *PtoMYB216*. The genomic DNA fragment containing a 1.8-kb upstream sequence of *PtoMYB216* was amplified by the primers: Pro-PtoMYB216 (F: 5′-ATGGAGGTGATCATGAAGCTTC-3′; R: 5′-GCTGCAATGCAGTGCTGGCA-3′). The *PtoMYB216*:GUS construct in the binary vector pCXGUS-P [32] was made by ligating the PCR-amplified genomic DNA fragments. The constructs were transformed into wild-type *Arabidopsis* plants by the floral dip method [35]. Transformants were selected on MS plates supplemented with 30 µg/mL of hygromycin and 50 µg/mL of carbenicillin. The first generation of 6-week-old transgenic plants was examined for the expression of the GUS reporter gene [36].

### RNA Extraction and RT-PCR Analysis

Total RNA was extracted from transgenic plants and untransformed control using TRIzol® Reagent (Invitrogen, Beijing, China) according to the manufacturer’s instructions. For reverse transcription (RT)-PCR, 2 µl RNA was reverse transcribed in a total volume of 20 µl, using PrimeScript™ RT reagent Kit with gDNA Eraser (Takara, Dalian, China) according to the manufacturer’s instructions. To detect *PtoMYB216* expression, PCR amplification was performed for 26 cycles, with each cycle consisting of 94°C for 1 min, 58°C for 30 s, respectively, 72°C for 1 min and finally 10 min at 72°C. The constitutively expressed *18S* gene was used to confirm that equal amount of cDNA in each reaction.

### Quantitative Real-time PCR

Total RNA extracted from leaves, roots, stems and petioles of poplar plants was reversed into cDNA using PrimeScript™ RT reagent Kit with gDNA Eraser (Takara, Dalian, China) according to the manufacturer’s instructions. The reverse transcribed cDNA samples used for real-time PCR, which was performed on a BioRad IQ5 real-time PCR detection system. The specific primers for *PtoMYB216* were qt-PtoMYB216-F: 5′-CATCTCAACATGTGTACAGTG-3′ and qt-PtoMYB216-R: 5′-GCAGATCCTTGGTATAGATGT-3′, and primers for *Populus 18S* gene were qt-18S-F: 5′-GGCATGGAAGGTGATGCAGATC-3′and qt-18S-R: 5′-CTGTGTCAAACAAGAACTTGTCC-3′. Quantitative real-time PCR reaction and data analysis were performed as described by Tsai et al [37] in a 25 µl reaction volume containing 12.5 µl of SYBR Premix ExTaq™ (TaKaRa, Dalian, China). Differences in gene expression, expressed as fold change relative to control, were calculated using the 2^ΔΔCT^ method. Each measurement was carried out in triplicate, and the error bars represent SE of the mean of fold changes for the three biological replicates. Analysis of lignin pathway genes was similarly performed using gene-specific primers (Table S1 in File S1).

### Subcellular Localization

The MYB216-GFP construct was created by ligating the PCR-amplified cDNA of *PtoMYB216* in the pCX-DG [32]. The expression vectors were induced into onion epidermal cells by Gene Gun (GJ-1000, SCIENTZ, China). The onion skin was stained with DAPI, and then photographed by confocal microscopy (Leica TCS SP5).

### Yeast Single-hybridization Assay

The yeast single-hybridization system was used with full-length transcription factors in pGBKT_7_ (Clontech) and introduced into the yeast strain *Saccharomyces cerevisiae* Gold2, as described previously [38]. Transformants were grown on SD medium lacking tryptophan (Trp) for positive clone selection and then on SD medium lacking Trp, histidine (His) and adenine (Ade) for the transactivation assay, according to the manufacturer’s instructions. X-α-gal was used to identify the transcription activation activity of PtoMYB216.

### Histology

Poplar petioles and stems were sectioned using a vibrating microtome to generate 100-µm serial sections. For histochemical analysis of lignification, sections were stained with 1% (w/v) phloroglucinol in 6 N HCl, viewed under the microscope [39].

### Determination of Lignin Content

Lignin content of stems was measured by modified Klason lignin method [40]. In brief, freeze-dried poplar was ground to pass a 40-mesh screen using a mill. The ground poplar (1 g) was then soxhlet-extracted with 100 ml acetone for 8 h to remove extractable components. The total weight of extractable components was determined gravimetrically by rotary evaporation. The extracted lignocellulosic material was air-dried to remove solvent and was then analyzed in triplicate for sugar and lignin composition as follows. A 0.2 g sample of extracted poplar was transferred to a 15-ml reaction vial in an ice bath. A 3-ml aliquot of 72% (w/w) H_2_SO4 was added to the sample and was thoroughly mixed for 1 min. The test tube was immediately transferred to a water bath maintained at 20°C and was subsequently mixed for 1 min after every 10 min. After 2 h of hydrolysis, the contents of each test tube were transferred to a 125-ml serum bottle, using 112 ml nanopure H_2_O, to rinse all residue and acid from the reaction vial. The serum bottles (containing 115 ml H_2_SO4 at 4% (w/w) plus poplar) were sealed with septa and autoclaved at 121°C for 60 min. Samples were allowed to cool, and the hydrolysates were vacuum-filtered through pre-weighed medium coarseness sintered-glass crucibles, washed with 200 ml warm (approximately 50°C) nanopure H_2_O to remove residual acid and sugars and dried overnight at 105°C. The dry crucibles were weighed to determine Klason (acid-insoluble lignin) lignin gravimetrically.

### Statistical Analysis

The Student’s *t* test program (http://www.graphpad.com/quickcalcs/ttest1.cfm) was used for statistical analysis of the data in the experiments of quantitative RT-PCR. In all these experiments, it was found that the quantitative differences between the two groups of data for comparison were statistically significant (*P*<0.001).

### Accession Numbers of MYBs from Different Species

The accession numbers of the MYB genes were: *PtrMYB170* (POPTR_0014s10680.1), *PtrMYB55* (POPTR_0014s10680.1), *PtrMYB121* (POPTR_0002s18700.1), *AtMYB83* (NM_111685), *EgMYB2* (AJ576023), *AtMYB46* (NM_121290), *PtMYB4* (AY356371), *AtMYB61* (NM_100825), *AtMYB103* (NM_124993), *AtMYB26* (Z95749), *AtMYB85* (NM_118394), *PtMYB1* (AY356372), *ZmMYB31* (NM_001112479), *ZmMYB42* (NM_001112539), *AtMYB58* (NM_101514), *AtMYB63* (Z95786).

## Results

### Isolation and Characterizaiton of the *PtoMYB216* Gene from *P. tomentosa* Carr

**Figure 1:**
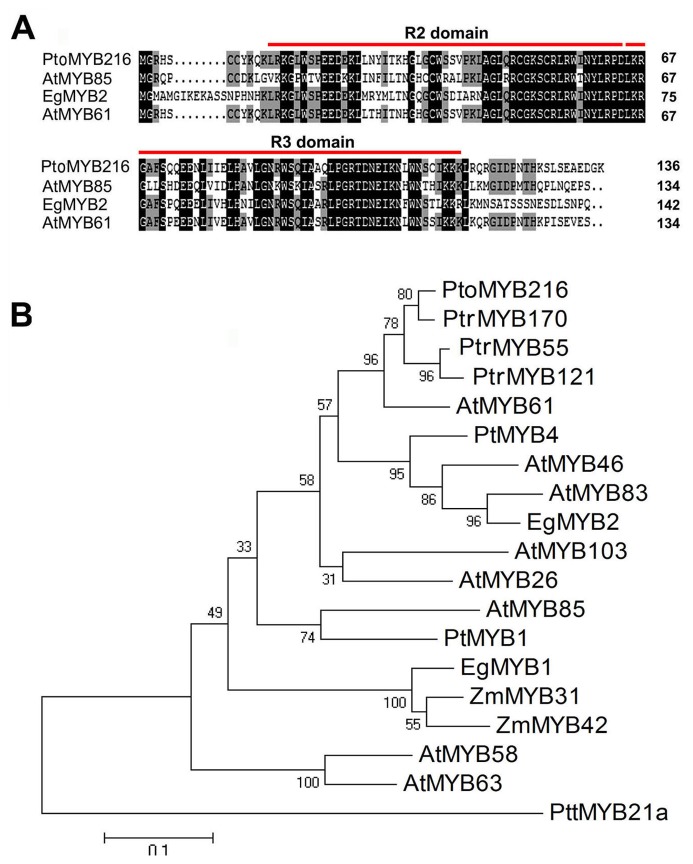
Sequence analysis of the *PtrMYB216* cDNA. (A) Sequence similarity of the N-terminal region of predicted PtrMYB216 including the conserved R2R3 DNA-binding domains with other MYB transcription factors. The R2R3 MYB domain is underlined. Identical and similar amino acid residues are shaded with black and gray, respectively. (B) Phylogenetic analysis of PtrMYB216 together with other MYBs involved in regulation of secondary wall biosynthesis or phenylpropanoid metabolism. Phylogenetic analysis was performed using the neighbor-joining method using MEGA version 4. Bar, 0.1 substitutions per site. Additional sequences include *Arabidopsis thaliana* (AtMYB26, At3g13890; AtMYB46, At5g12870; AtMYB58, At1g16490; AtMYB61, At1g09540; AtMYB63, At1g79180; AtMYB83, At3g08500; AtMYB85, At4g22680; AtMYB103, At1g63910), *Zea mays* (ZmMYB31, CAJ42202; ZmMYB42, CAJ42204), *Eucalyptus gunnii* (EgMYB1, CAE09058; EgMYB2, AJ576023), *Pinus taeda* (PtMYB1, AY356372; PtMYB4, AY356371), *Populus tremula* × *tremuloides* (PttMYB21a, AJ567345), *P. trichocarpa* (PtrMYB216, POPTR_0013s00290.1).

We isolated the *PtoMYB216* cDNA encoding a putative R2R3 MYB protein, by RT-PCR using gene-specific primers based on the sequences deposited in the *Populus* genome database. *PtoMYB216* appeared to be a full-lengh cDNA of 1290 bp encoding a protein of 423 amino acids residues. *PtoMYB216* exhibits close sequence similarity to *PtrMYB216* from *P. trichocarpa*. The amino-teriminal extremity of PtoMYB216 contains a typical R2R3 inperfect repeats responsible for binding to target DNA sequences and is highly conserved among R2R3-MYB proteins (Fig. 1A). Comparison of the *PtoMYB216* sequence with databases showed high identity with lignin-associated MYB proteins from *Arabidopsis* (AtMYB61, 87.50%; AtMYB85, 62.50%), *Eucalyptus gunnii* (EgMYB2, 61.11%) (Fig. 1A). Previously, a number of MYB transcription factors have been reported to be involved in regulation of phenylpropanoid biosynthesis, secondary xylem development, or secondary wall formation [5], [22], [25]–[27], [41]. Phylogenetic analysis demonstrated that *PtoMYB216* has a close relationship with AtMYB46, AtMYB61, AtMYB83 and EgMYB2 (Fig. 1B), indicating it is a potential transcriptional activator in secondary wall biosynthesis.

### The Expression Profiles of *PtoMYB216* in Poplar Tissues

**Figure 2:**
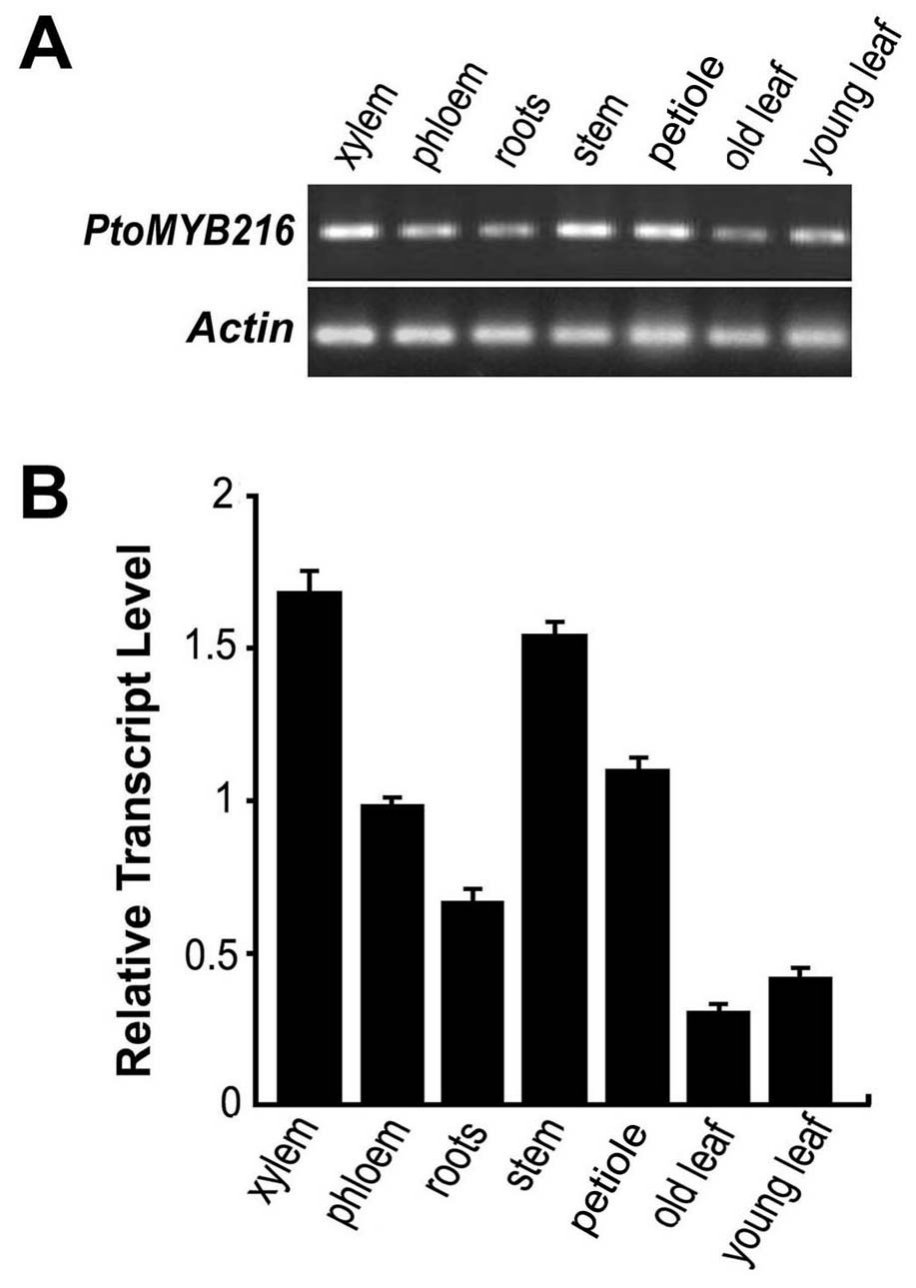
Expression patterns of *PtoMYB216* in various tissues of *P*. tomentosa Carr. Semi-quantitative RT-PCR analysis of *PtoMYB216* expression in various tissues of *P*. tomentosa Carr. (B) Quantitative real-time PCR analysis of *PtoMYB216* transcript levels in various tissues of *P*. tomentosa Carr. *Actin* expression was used as a control. Total RNA was isolated from xylem, phloem, root, stem, petiole, old leaf, young leaf.

*PtoMYB216* expression in various tissues of Chinese white poplar was analyzed by semiquantitative reverse transcription (RT)-PCR (Fig. 2A). Expression was detected in all tissues tested with the highest mRNA level in xylems. PtoMYB216 transcripts were present at very low levels in young and old leaves. Quantitative real-time PCR analysis with different tissues further showed that the relative levels of the *PtoMYB216* transcripts in the xylems were more than twice as abundant as in the phloems and were 2-fold higher than in the roots (Fig. 2B). This expression profile in lignin-rich tissue, which is in agreement with the fact that *PtoMYB216* had been cloned from a cDNA library of Chinese white poplar xylem, suggest that *PtoMYB216* is involved in the regulation of lignin biosynthesis. Taken together, expression of the *PtoMYB216* gene is not xylem specific but is clearly up-regulated during secondary wall formation.

### Promoter Analysis of *PtoMYB216* in *Arabidopsis*

**Figure 3:**
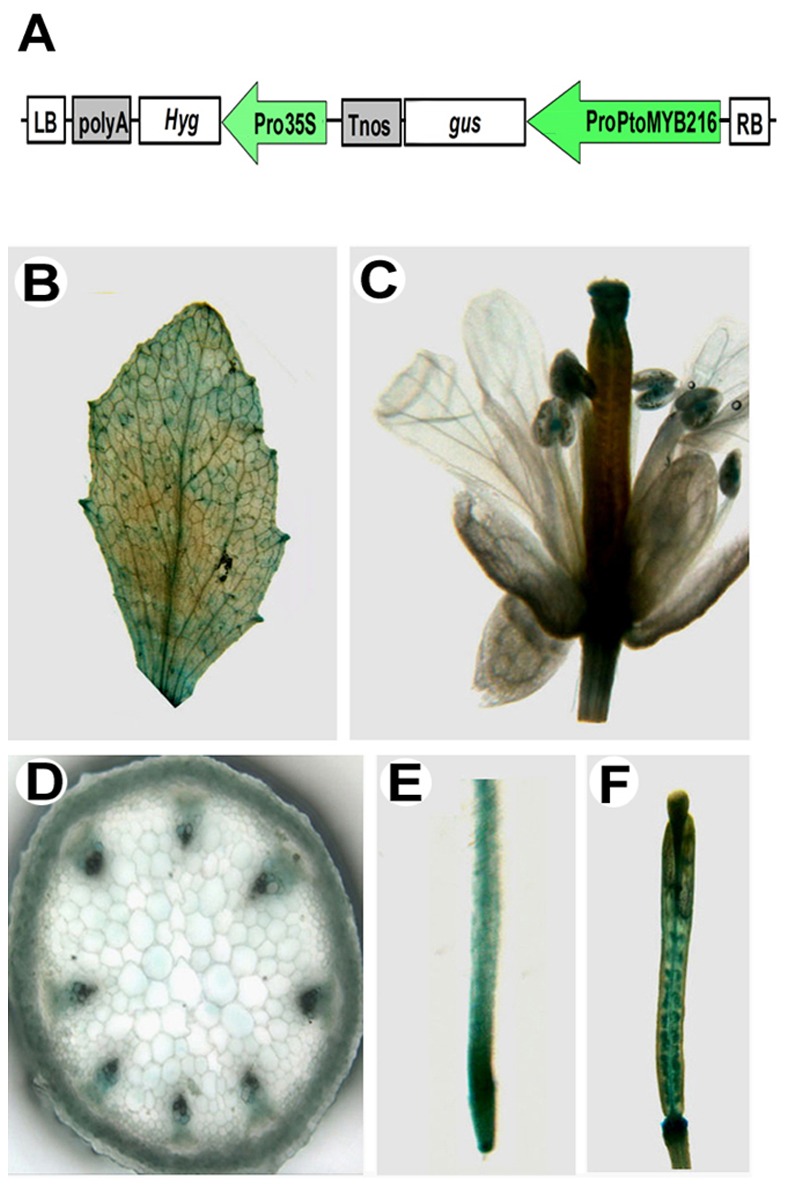
Expression analysis of *PtoMYB216* gene promoters. *PtoMYB216* promoter-driven GUS construct was generated (A) and introduced into *Arabidopsis thaliana*. Transgenic seedlings were grown on MS media and assayed for GUS activity. GUS expression was observed in various tissues of *PtoMYB216*::*GUS* plants, including leaf (B), flower (C), root (E), silique (F) and cortex and in vascular bundles in the cross section of stem (D).

To further investigate the spatial and temporal expression pattern, a vector containing the uidA coding sequence (as reporter gene) under the control of the 1.8-kb promoter region of *PtoMYB216* was introduced into wild-type *A. thaliana* plants. Histochemical analysis of GUS expression in the progeny of transformed plants showed that the *PtoMYB216* promoter drove the GUS expression in the vascular region of all stained organs (roots, leaves, flowers and siliques). Stem sections revealed the GUS expression in protoxylem, metaxylem, intrafascicular cambium and fibers (Fig. 3). This restricted pattern of expression demonstrated that the *PtoMYB216* gene was expressed in tissues undergoing secondary cell wall thickening.

### Subcellular Localization and Transcriptional Activation Activity of *PtoMYB216*

**Figure 4:**
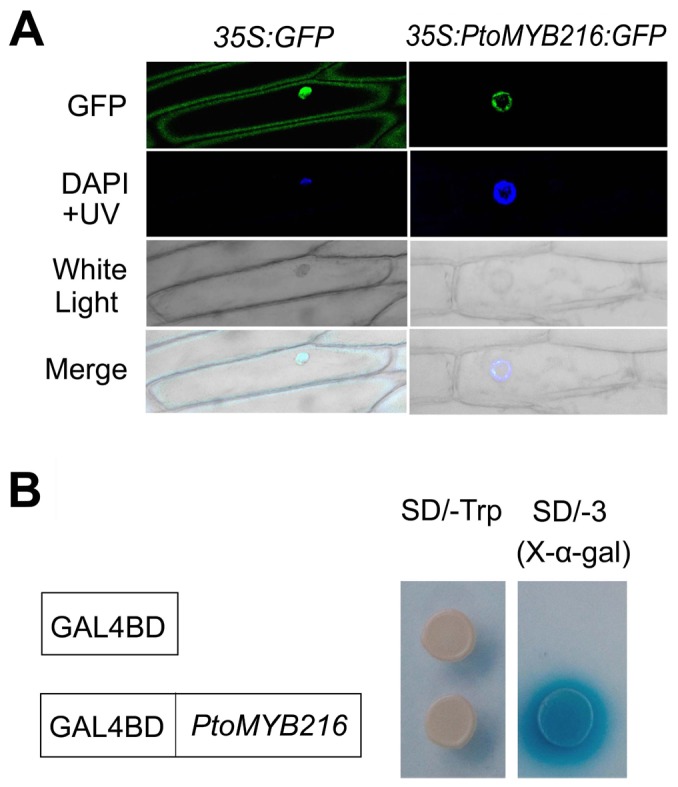
Nuclear localization and transcriptional activity of PtoMYB216. (A) Onion epidermis was transformed with *35S:PtoMYB216:GFP* and 35S:GFP constructs by particle bombardment. GFP fluorescent images were examined with confocal microscope at 18 h after bombardment. The position of nuclei was confirmed by DAPI staining and bright-filed images were compared. (B) Transcriptional activation analysis of PtoMYB216 ORF fused with the GAL4 DNA-binding domain (GAL4BD) showing its ability to activate the expression of the Trp and α-Gal reporter genes in yeast.

We assayed the subcellular localization of a PtoMYB216-green fluorescent protein (GFP) fusion in onion epidermal cells and found that the fusion protein accumulated in the nucleus (Fig. 4A), consistent with the predicted function as transcription factors. To examine whether PtoMYB216 is transcriptional activators, we fused it with the GAL4 DNA-binding domain for transactivation analysis in yeast. It was shown that *PtoMYB216* was able to induce the expression of the *LacZ* reporter gene (Fig. 4B), indicating that it is a transcriptional activator.

### Overexpression of *PtoMYB216* in *P. tomentosa* Carr

**Figure 5:**
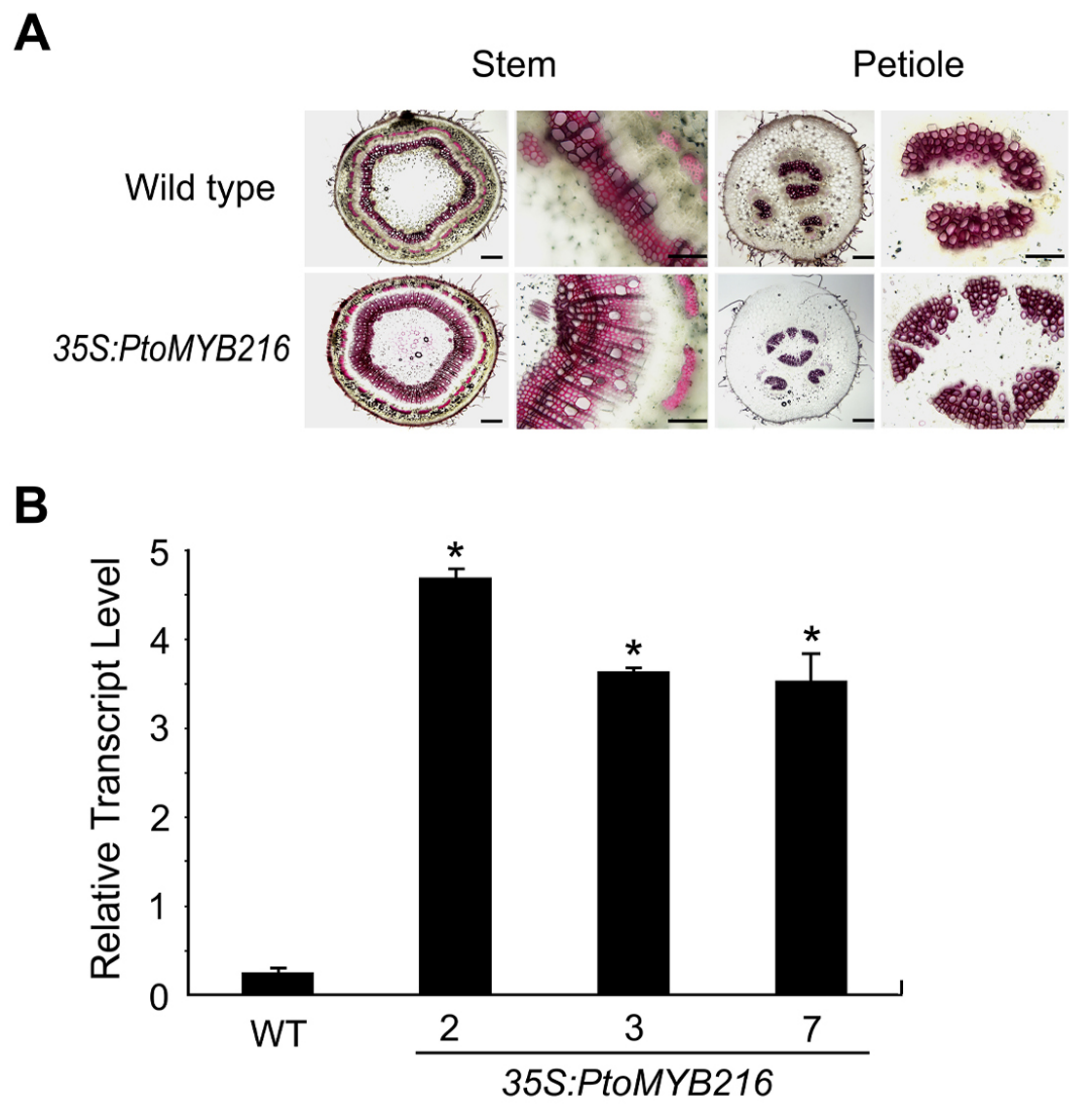
Ectopic expression of *PtoMYB216* results in an increase of xylem secondary cell wall thickness. (A) View of vascular tissues stained by Phloroglucinol-HCl in transverse sections of stem and petiole of the *35S:PtoMYB216* plants and the wild-type. (B) Quantitative real-time PCR analysis of *PtoMYB216* transcript levels in stems of transgenic poplar plants. WT, the wild type; 2, 3, and 7, *PtoMYB216*-overexpressing lines. Error bars represent SE of three replications.

To investigate whether *PtoMYB216* is involved in the regulation of secondary wall biosynthesis, the *PtoMYB216* open reading frame under the control of the cauliflower mosaic virus 35S promoter was introduced into Chinese white poplar by *A. tumefaciens*-mediated transformation. A total of 10 hygromycin-resistant putative transformants were obtained and grown in the greenhouse. The generated transgenic plants did not show any phenotypic changes compared with wild-type plants, except for a slight reduction in plant height (Figure S1 in File S1). PCR analysis using gene-specific primers showed that an expected amplification product specific for *Hpt* was obtained from all transgenic lines tested, whereas no signal was detected from wild-type plants (Figure S2 in File S1), confirming the integration of the transgene into the poplar genome. From all of the independent hygromycin-resistant transgenic lines harboring the *35S:PtoMYB216* construct, three independent lines (2, 3 and 7) with high *PtoMYB216* transcript levels were selected for further analysis (Fig. 5B).

To determine lignin localization in *35S:PtoMYB216* and control poplar plants, petiole and stem sections were stained with phloroglucinol-HCl, which reacts specifically with lignins to form a red chromophore. As expected, phloroglucinol-HCl stained the tissues of the wild type lines less intensely than the 3*5S:PtoMYB216* tissues (Fig. 5A). In stem sections, lignins were observed in the xylems of the control plants, while much higher concentration of lignins was detected in the xylems of the *PtoMYB216*-overexpressing plants (Fig. 5A). In petioles, staining was observed only in the xylems in the controls, while in *35S:PtoMYB216* plants, *PtoMYB216* overexpression caused ectopic deposition of lignin (Fig. 5A). The deposition of lignin in stems of *PtoMYB216* overexpressors was confirmed by chemical analysis of lignin composition. The results showed that Klason lignin content in stems of *35S:PtoMYB216* plants was almost 2-fold higher than that of the wild type (Table 1), demonstrating that *PtoMYB216* is capable of activating the lignin biosynthetic pathway.

**Table 1:** Lignin content in the stems of the wild type and transgenic poplar overexpressing *PtoMYB216* as determined by Klason analysis.

Lines	Lignin(mg/100 mg)
Wild type	20.68±1.42
*35S:PtoMYB216* Line 2	29.14±2.43
*35S:PtoMYB216* Line 3	25.81±0.85
*35S:PtoMYB216* Line 7	28.42±1.76

The analysis reveals a higher lignin content in *PtoMYB216* overexpressor to the wild type. Each data point is the mean (mg/100 mg dry stems) ± SE of three separate assays.

### Induction Expression of Genes Associated with Secondary Cell Wall Biosynthesis by *PtoMYB216* Overexpression

**Figure 6:**
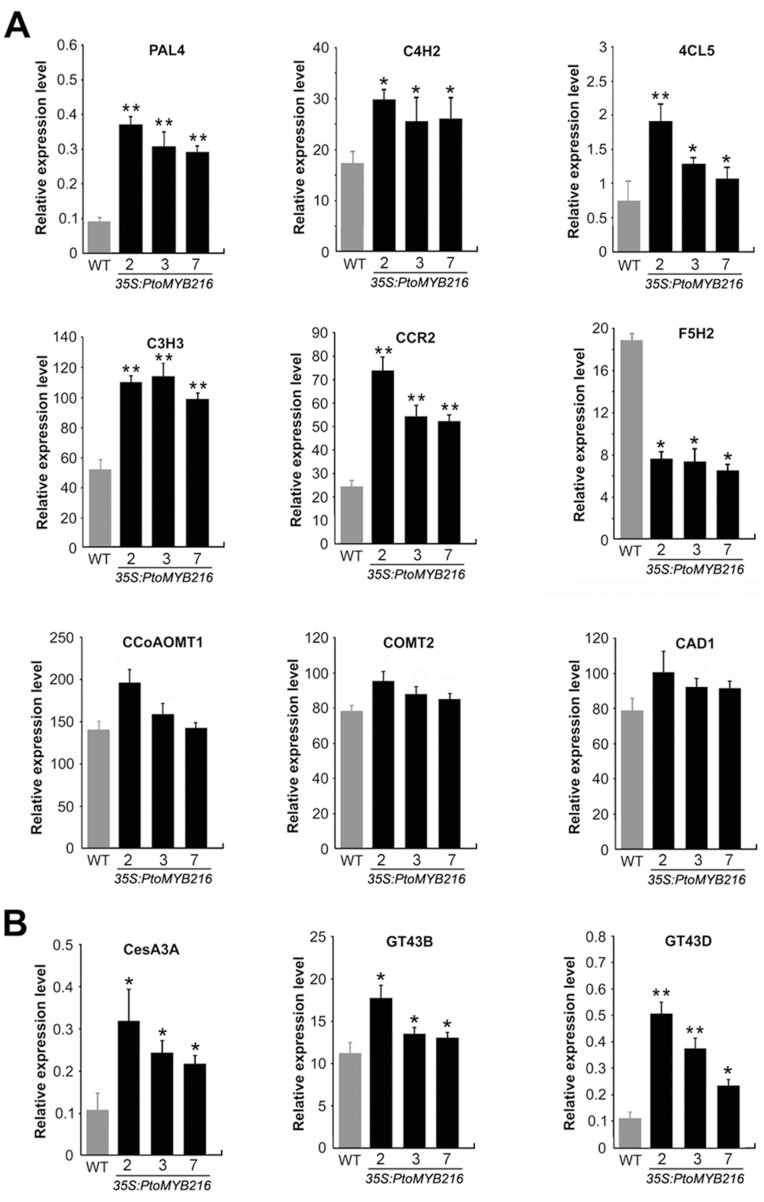
Gene expression analyses of lignin biosynthetic genes in transgenic plants overexpressing *PtoMYB216*. Transcript accumulation was quantified by quantitative real-time polymerase chain reaction (qRT-PCR). The quantitative differences of the expression of the tested genes between the wild type and the overexpression lines are statistically significant (P<0.05). Error bars represent SE of three biological replicates. (A) Overexpression of *PtoMYB216* induces the expression of lignin biosynthetic genes. The genes selected for expression analysis were previously shown to be highly expressed in stems where lignified fibers and vessels are abundant [51]. PAL4, phenylalanine ammonia lyase 4; C4H2, cinnamate 4-hydroxylase 2; 4CL5, 4-coumarate CoA ligase 5; C3H3, p-coumarate 3-hydroxylase 3; CCoAOMT1, caffeoyl CoA 3-O-methyltransferase 1; CCR2, cinnamoyl CoA reductase 2; F5H2, ferulate 5-hydroxylase 2; COMT2, caffeic acid O-methyltransferase 2; CAD1, cinnamyl alcohol dehydrogenase 1. (B) Overexpression of *PtoMYB216* induces the expression of secondary wall-associated cellulose synthase genes (CesA2B, CesA3A), xylan biosynthetic genes (GT43B and GT43D).

Since manipulation of *PtoMYB216* function resulted in an increase in lignin content, we used qRT-PCR with gene specific primers (Table S1 in File S1) to investigate the expression of genes encoding enzymes involved in the general phenylpropanoid metabolism as well as in the specific branch toward monolignol biosynthesis. All of the upsteam genes including L-phenylalanine ammonia-lyase gene (*PAL4*), 4-hydroxycinnamate:CoAligase gene (*4CL5*), ρ-coumar-oylshikimate 3′-hydroxylase gene (*C3*′*H3*) and cinnamoyl-CoA reductase gene (*CCR2*) were found to be up-regulated in the transgenic plants overexpressed *PtoMYB216* (Fig. 6A). Interestingly, the expression of ferulate 5-hydroxylase gene (*F5H2*) was significantly decreased in the *PtoMYB216* overexpressors, but no obvious changes in transcript levels of caffeoyl-CoA O-methyltransferase gene (*CCoAOMT1*), O-methyltransferase gene (*COMT2*) and cinnamyl alcohol dehydrogenase gene (*CAD1*) were observed (Fig. 6A). We also assayed the expression of genes associated with cellulose biosynthesis and found that overexpression of *PtoMYB216* resulted in an elevation in the expression of *CesA3A* [42], and two xylan biosynthetic genes *GT43B* and *GT43D* [42] (Fig. 6B). Taken together, these results demonstrate that *PtoMYB216* is transcriptional activators of lignin biosynthesis during secondary wall formation.

## Discussion

Lignin is the second most abundant biomass component produced by vascular plants. The lignin polymers are mainly deposited in secondary wall–containing cells, such as fibers and tracheary elements, and provide plants with structural support, a mechanical barrier against pest attack, and the raw material for constructing the vascular system [43], [44]. Most previous molecular and genomic studies on wood formation have involved in major efforts on the identification of the biosynthetic genes of lignin polymers, which is the first important step toward our understanding of wood formation and our attempts to genetically modify wood quantity and quality [45]. In addition to genes encoding enzymes involved in lignin biosynthesis, recently many transcription factors have been shown to regulation of lignin biosynthetic pathway [16]. Most lignin activators characterized to date are from the MYB family. Here, we have characterized a new R2R3 MYB gene (*PtoMYB216*) from *P. tomentosa* Carr. and preferentially expressed in secondary xylem (Fig. 2). Subcellular localization analysis showed that PtoMYB216 protein targeted to the nucleus and a transactivation assay of this gene obtained positive results (Fig. 3), indicating that PtoMYB216 works as a transcription factor. Overexpression of *PtoMYB216* in poplar resulted in an ectopic deposition of lignin (Fig. 5) and activation of lignin biosynthetic genes (Fig. 6).

The R2R3-MYB proteins comprise one of the largest families of transcription factors in plants. R2R3-MYB family members play central roles in plant-specific processes, such as the elaboration of specialized cell types, including xylem, guard cells, trichomes, and root hairs, and the biosynthesis of specialized branches of metabolism, including lignin biosynthesis [46]. In a previous study, the entire *P. trichocarpa* MYB family has been characterized and 192 R2R3-MYB genes in the *P. trichocarpa* genome were annotated [30]. Phylogenetic analysis of the predicted R2R3-MYB protein sequences reveals that these proteins are clustered into 49 subgroups, and clade 10 includes four R2R3-MYB family members, PtrMYB2, PtrMYB3, PtrMYB21and PtrMYB20, implicated in the regulation of genes encoding lignin biosynthetic enzymes [30]. This clade also includes *Pinus taeda* MYB4 [21], AtMYB46 [23], AtMYB61 [47], *Eucalyptus gunnii* MYB2 [22], and *Picea glauca* MYB4 [31]. These genes have been shown to function in an almost identical fashion, with expression in differentiating xylem cells and capacity to alter lignin biosynthesis. Recently, it is demonstrated that the poplar wood-associated MYB transcriptional activators PtrMYB3 and PtrMYB20, activate the biosynthetic pathways of cellulose, xylan and lignin when overexpressed in *Arabidopsis* and they are also able to activate the promoter activities of poplar wood biosynthetic genes [29], suggesting their involvement in the regulation of wood formation in poplar. In our phylogenetic analysis, PtoMYB216 protein groups in this lignification-related R2R3-MYB clade and it is most similar to AtMYB61 from *A. thalian* (Fig. 1A). A previous study has demonstrated that overexpression of AtMYB61 causes ectopic lignification and phenocopies a dark-photomorphogenic mutant, *de-etiolated 3* (*det3*) [41]. AtMYB61 also controls stomatal aperture in *Arabidopsis* [48] and seed coat mucilage deposition [49], indicating that AtMYB61 might function as a coordinate regulator of multiple and distinct biosynthetic pathways. In this study, *PtoMYB216* overexpresison induced cell wall modifications in xylem of transgenic plants, suggesting that PtoMYB216 plays a role in regulating lignin biosynthesis in poplar. Additionally, lignin also plays a role in plant responses to biotic and abiotic stresses [50]. For example, rice plants over-expressing *OsWRKY89* showed enhanced tolerance to the rice white-backed planthopper *Sogatella furcifera*, associated with increase in lignification of stems [4]. It is still unknown whether *PtoMYB216* also play regulatory roles in other biosynthetic pathways. Therefore, future work will focus on understanding the function and regulation of this gene in biotic and abiotic stress responses.

Previous studies have shown that R2R3 MYB transcription factors are involved in directly regulating secondary cell wall formation and lignin deposition by binding to AC elements in the promoters of lignin biosynthetic pathway genes [5], [21], [22]. The biochemical pathways of monolignol biosynthesis are highly conserved throughout vascular plants, and most of the enzymes in the monolignol biosynthesis pathway have been identified and characterized [5]. AC elements have been found in the promoters of *PAL*, *4CL*, *C3H*, *CCoAOMT*, *CCR* and *CAD* [51]. In the present study, we found that overexpression *PtoMYB216* only activated the expression of the upstream genes (*PAL4*, *4CL5*, *C4H2* and *C3H3*) in the lignin biosynthetic pathway. Whereas *CCoAOMT* expression was not changed although AC elements are present in its promoter (Fig. 6). It is most likely that PtoMYB216 just binds to the AC elements in the promoters of these upstream genes in the lignin biosynthetic pathway in poplar. C4H, F5H and COMT have not apparent AC elements in their promoters [51], although it is possible that more degenerate AC elements may be present in their promoters. Consistent with this possibility, the *C4H2* gene is also shown to be directly activated by *PtoMYB216* (Fig. 6).

MYB transcription factors act as activators of lignin biosynthesis. For example, overexpression of pine *PtMYB4* in tobacco led to increased lignin content due to enhanced expression of *C3H*, *CCOMT*, *COMT*, *CCR* and *CAD* genes [21]. Lignin quality can also be controlled by MYB transcription factors. An increased S/G ratio without any change in lignin content was found in transgenic tobacco overexpressing eucalyptus *EgMYB2* and the expression of the downstream genes in lignin biosynthetic pathway was highly induced in these plants [22]. Newman et al. [41] has previously reported that overexpression of *AtMYB61*, a homolog of *PtoMYB216*, causes ectopic lignification in *Arabidopsis*. Here, we found that *PtoMYB216* overexpression in poplar plants resulted in an increase in lignin content (Table 1), while no impact on the S/G ratio of the lignin (Data not shown). qRT-PCR analysis showed that expression of *CCoAOMT1*, *COMT2* and *CAD1* were not changed in transgenic plants overexpressing *PtoMYB216* (Fig. 6). It is consistent with the reported results, in which the transcription of *CCoAOMT1*, *COMT2* and *CAD1* was modulated according to the metabolic demand of monolignol biosynthesis.

*PtoMYB216* overexpression is sufficient to induce lignification, but it remains to be determined if it is necessary for lignification. In general, it is the possible functional redundancy in the R2R3-MYB family in plants. In *P. tomentosa* Carr., there are at least four R2R3 MYB family members (*PtoMYB170*, *PtoMYB55*, *PtoMYB121* and *PtoMYB216*) that could be the functional orthologues *AtMYB61* (Fig. 1B). Such functional redundancy confounds loss-of-function experiments aimed at determining necessity. In gene families that have functional redundancy, related family members often must be suppressed before a phenotype is observed [52]. In order to address these problems in future experiments aimed at testing necessity of the *PtoMYB216* homologs to regulate lignification, it may be necessary to suppress several MYB family members simultaneously, focusing on the *Arabidopsis AtMYB61*orthologues.

## Supporting Information

File S1 **Contains: Table S1 List of primers used for qRT-PCR. Figure S1. Photographs of** ***PtoMYB216*** **overexpressing** ***Populus tomentosa*** **Carr. after four weeks of growth.** No phenotypic or growth differences were observed between *PtoMYB216* overexpressing and control plants under the same growth conditions. **Figure S2. PCR analysis of transgenic poplar plants. Genomic DNAs were isolated from hygromycin-resistant plants transformed with the** ***35S*** **:** ***PtoMYB216*** **vector.** PCR amplification using primers specific for the production of a 562-bp *PtoMYB216* fragment. M, D2000 DNA Ladder; WT, non-transgenic plants; P, corresponding plasmid DNA (positive control); Lanes 1–10, independent transgenic lines. Numbers on the left indicate DNA marker sizes in base pairs.
(DOC)
