# Supplementary material for: Functional Characterization of the Poplar R2R3-MYB Transcription Factor PtoMYB216 Involved in the Regulation of Lignin Biosynthesis during Wood Formation
Source: PLoS One. 2013 Oct 28;8(10):e76369. doi: 10.1371/journal.pone.0076369 (PMC3810269; doi:10.1371/journal.pone.0076369)
Supplement: File S1 — Contains: Table S1 List of primers used for qRT-PCR. Figure S1. Photographs of PtoMYB216 overexpressing Populus tomentosa Carr. after four weeks of growth. No phenotypic or growth differences were observed between PtoMYB216 overexpressing and control plants under the same growth conditions. Figure S2. PCR analysis of transgenic poplar plants. Genomic DNAs were isolated from hygromycin-resistant plants transformed with the 35S : PtoMYB216 vector. PCR amplification using primers specific for the production of a 562-bp PtoMYB216 fragment. M, D2000 DNA Ladder; WT, non-transgenic plants; P, corresponding plasmid DNA (positive control); Lanes 1–10, independent transgenic lines. Numbers on the left indicate DNA marker sizes in base pairs. (DOC) [file pone.0076369.s001.doc]

**Functional characterization of the poplar R2R3-MYB transcription factor PtoMYB216 involved in the regulation of lignin biosynthesis during wood formation**

Qiaoyan Tian^1, #^, Xianqiang Wang^1, #^, Chaofeng Li^1, #^, Wanxiang Lu^1,2^, Li Yang^1^, Yuanzhong Jiang^1^, Keming Luo*^1,3^

**-------------------------------------------------------------------------------------------------------**

^1-^Key Laboratory of Eco-environments of Three Gorges Reservoir Region, Ministry of Education, Chongqing Key Laboratory of Transgenic Plant and Safety Control, Institute of Resources Botany, School of Life Sciences, Southwest University, Chongqing 400715, China

^2-^College of Horticulture and Landscape Architecture, Southwest University, Chongqing, 400716, China

^3-^Key Laboratory of Adaptation and Evolution of Plateau Biota, Northwest Institute of Plateau Biology, Chinese Academy of Sciences, 810008 Xining, China

**Supporting Information Legends**

**File S1** Table S1 List of primers used for qRT-PCR.

| **Sequence name** | **GenBank** | **Primers** |
| --- | --- | --- |
| *PAL4* | XM_002322848 | Forward: 5’-CCTACATTGACGATCCTTGCAG-3’ |
|  |  | Reverse: 5’-GACCTGCATTCCTTGATCCTG-3’ |
| *4CL5* | EU603299 | Forward: 5’-CATCCGAGGTGATCAGATCATG-3’ |
|  |  | Reverse: 5’-CACAGCAGCATCAGATATCC-3’ |
| *C3H3* | XM_002336315 | Forward: 5'-GAGGTTCCTGGAGGAGGATGG-3' |
|  |  | Reverse: 5'-GGAGTCGTCATGTAAGTGAC-3' |
| *CCR2* | XM_002332044 | Forward: 5’-CTGTTCAAGCTTATGTGCATG -3’ |
|  |  | Reverse: 5’-GTGGAGAACGCTCTCAGAGC -3’ |
| *C4H2* | JX550321 | Forward: 5'-GAGCAAGATCCTGGTAAACGC-3' |
|  |  | Reverse: 5'-CTGAGGTGTCAATCTTGGACTG-3' |
| *CCoAOMT1* | EU603307 | Forward: 5’-CAAGAGGTTGATTGAGCTTG -3’ |
|  |  | Reverse: 5’-GGTCAGCAGCAAGTGCCTTG -3’ |
| *COMT2* | JX552702 | Forward: 5’-CATGAAGTGGATATGCCATG -3’ |
|  |  | Reverse: 5’-GTTGAATGCACAGCACATTAC -3’ |
| *F5H2* | HQ613769 | Forward: 5'-GAGTCCAGCAAGAGCTCGCAG-3' |
|  |  | Reverse: 5'-GCATAAGCATTGATCATCAC-3' |
| *CAD1* | EU603306 | Forward: 5’-CAAGCTGATCTTGATGGGTG -3’ |
|  |  | Reverse: 5’-CGAATCTATATCTCACATC -3’ |
| *CesA3A* | JX552264 | Forward: 5’-GTCCTTGGAGGAGTAGACAC -3’ |
|  |  | Reverse: 5’-GGATAGAGATGGACAATGAC -3’ |
| *GT43B* | JF518935 | Forward: 5’-CTCAATCCTCTGGGATCCTG -3’ |
|  |  | Reverse: 5’-GAATTGTAATCTGCTCATC -3’ |
| *GT43D* | JF518937 | Forward: 5’-CTATGGTAGAGCCACTTGG -3’ |
|  |  | Reverse: 5’-GTAGAAACCTGTGTCTCCAC -3’ |

**File S1 Figure S1 Photographs of *PtoMYB216* overexpressing *Populus tomentosa* Carr. after four weeks of growth.** No phenotypic or growth differences were observed between *PtoMYB216* overexpressing and control plants under the same growth conditions.

**File S1 Figure S2 PCR analysis of transgenic poplar plants. Genomic DNAs were isolated from hygromycin-resistant plants transformed with the *35S*:*PtoMYB216* vector.** PCR ampliﬁcation using primers speciﬁc for the production of a 562-bp *PtoMYB216* fragment. M, D2000 DNA Ladder; WT, non-transgenic plants; P, corresponding plasmid DNA (positive control); Lanes 1–10, independent transgenic lines. Numbers on the left indicate DNA marker sizes in base pairs.
